# Fe(II) Addition Drives Soil Bacterial Co-Ocurrence Patterns and Functions Mediated by Anaerobic and Chemoautotrophic Taxa

**DOI:** 10.3390/microorganisms10030547

**Published:** 2022-03-02

**Authors:** Chenyang Zhang, Senlin Liu, Sarfraz Hussain, Lifeng Li, Baiome Abdelmaguid Baiome, Shuiqing Xiao, Hui Cao

**Affiliations:** 1 Key Laboratory of Agricultural Environmental Microbiology, Ministry of Agriculture and Rural Affair, College of Life Sciences, Nanjing Agricultural University, 6 Tongwei Road, Nanjing 210095, China; zhangchenyang@stu.jiangnan.edu.cn (C.Z.); senlin.liu@warwick.ac.uk (S.L.); sarfarazhussainkhosa@yahoo.com (S.H.); lilf@geneplus.org.cn (L.L.); biome_ali@yahoo.com (B.A.B.); 2 Wellington Research Group, School of Life Sciences, University of Warwick, Gibbet Hill Road, Coventry CV4 7AL, UK; 3 School of Intercultural Studies, Jiangxi Normal University, Nanchang 330022, China; echoxiaoshuiqing@163.com

**Keywords:** Fe(II), paddy soil, bacterial community structure, anaerobic bacteria, chemoautotrophic bacteria, co-occurrence networks

## Abstract

Iron is among the most abundant elements in the soil of paddy fields, and its valence state and partitioning can be transformed by flooding and drainage alternations. However, little is known about the function of soil microbes that interact with Fe(II). In this study, sandy and loamy soils originating from rice fields were treated with Fe(II) at low and high concentrations. The findings demonstrate that additional Fe(II) has various effects on the soil’s microbial community structure and metabolic pathways. We conclude that Fe(II) at high concentrations reduced bacterial abundance and diversity in two textured paddy soils, yet the abundance in loamy soils was higher than it was in sandy soil. Additionally, in environments with high Fe(II) levels, the relative abundance of both anaerobic and chemoautotrophic bacteria increased. The Fe(II) concentration was positively correlated with total reduced substances but negatively correlated with redox potential and pH. Co-occurrence networks revealed that Fe(II) significantly promoted interactions with the most anaerobic and chemoautotrophic bacteria. In addition, adding Fe(II) greatly increased the number of more complex bacterial networks, and an increase in the number of mutually beneficial taxa occurred. We found that Fe(II) promoted the methane pathway, the Calvin cycle, and nitrate reduction to small but significant extents. These pathways involve the growth and interrelation of autotrophic and anaerobic bacteria. These results suggest that changes in the bacterial community structure occur in many dry–wet alternating environments.

## 1. Introduction

Rice is one of the world’s three major food crops, and approximately 90% of paddy soil is distributed in Asia, where it is mainly concentrated in the subtropical and tropical regions [1]. Paddy soil is one of the most important soil types in China, with a planting area of 3.07 × 10^7^ hectares in 2017, accounting for 18.5% of the total sown area of crops [2]. The soil changes periodically under oxic and anoxic conditions through repeated flooding and drainage cycles [3]. These involved physical, chemical and biological processes have significantly impact paddy soil [4].

As the fourth most abundant element in the Earth’s crust, iron (Fe) is relatively abundant in many cultivated soils [5], and it is also one of the most commonly used redox-active metals. Further research has shown that most living organisms need transition metals to survive and metal toxicity can be increased by catalytic activity, we must strictly control their concentrations based on standards [6]. Fe(III) can serve as an electron acceptor for FeRB (Fe-reducing bacteria) that mineralize organic substances, and the redox potential between the Fe(III)/Fe(II) coupled reactions may support a large part of the microbial biomass. FeOB (Fe-oxidizing bacteria) play a vital role in the redox cycle of iron in both oxic and anoxic environments [7,8]. It has been reported that the biological oxidation of ferrous iron is possible under anoxic conditions because nitrate has light-independent chemotrophic microbial activity as an electron acceptor [9]. Microbial Fe oxidation not only can significantly affect the geochemistry of hydromorphic soils [5] and rice yield [10] but also is closely related to the biogeochemical cycles of carbon, nitrogen, nutrients, and heavy metals [11,12]. With the gradual formation of anaerobic conditions, immediate and thorough soil submergence will reduce the gas exchange between the soil and the air, causing the Fe(II) content to increase with Fe(III) complex reduction [13]. Fe(II) concentrations in paddy soils vary with flooding depth and duration [14]. As an essential micronutrient, Fe(II)’s participation in some life-sustaining processes of microbes and plants has been well documented. However, its role in shaping bacterial diversity in paddy soils and ecological function are not yet fully understood [15,16].

Soil texture is an essential physical soil property. Different soil texture types directly determine soil aeration, water retention, soil fertility, and farming difficulty. It has conclusively been shown that, compared with sandy soils, loamy soils have a greater buffering capacity for extremely acidic coal leachate containing heavy metals at high concentrations [17]. Some researchers also tested the effects of olive mill wastewater on four different textured soils, indicating that loamy soils also have a greater buffering capacity than sandy soils [18]. Recent research found that when biochar was added to soils, sandy soils were more sensitive than loamy soils, which may be due to the lower amount of organic and inorganic colloids in sandy soils [19]. Rather than just being isolated in communities, soil microbes exist in far more complicated systems [20]. Hitherto, most approaches for soil bacteria attempt to understand microbial diversity and how they adjust according to environmental factors, but few studies explain the connections among soil bacteria [21]. This network analysis provides new insights into how various microbes interact in communities. It is widely known that the co-occurrence among microbial operational taxonomic units (OTUs) or genera is derived from the molecular ecological network [22,23]. It is found that high copper concentrations in swine manure increased bacterial stability. Apart from this, he proposed that the coexistence of copper resistance genes (CRGs) and the bacterial community made substantial contributions to ecological networks [24]. However, little is known about the interactions of bacteria found in paddy soil systems after Fe(II) is added.

In this study, we designed various concentrations of Fe(II) in laboratory-simulated experiments on loamy and sandy paddy soil in southern China. Next, we applied qPCR and 16S rRNA amplicon sequencing for further research. We hypothesized that the addition of Fe(II) would change the bacterial community and function of paddy soil and that the effect varies with the soil texture. In addition, we hypothesized that the addition would trigger the competitive interaction of key microbiota in the bacterial network. The study aims to (1) elucidate the responses of soil bacterial communities and anaerobic and chemoautotrophic taxa to Fe(II) and soil texture and (2) reveal the changes in soil bacterial functions and ecological networks.

## 2. Materials and Methods

### 2.1. Site Description and Soil Sampling

Two distinct textured soils (loamy and sandy soils) were derived from two paddy fields (30°50′8.4″ N, 118°36′21.9″ E and 30°49′33″ N, 118°54′47″ E) located in Xuanzhou District of Anhui Province, southern China, which is a critical rice-growing region of China. The soil samples were collected during December, 2015. Soil samples were taken from the top layer (0–20 cm) of the litter, wood, and unwanted material. Approximately 10 kg of each soil was collected. Soil samples were thoroughly mixed and then air-dried at room temperature for 24 h. Visible plant detritus, stones, and other extraneous matter were removed, and then the samples were sieved with a 2 mm mesh sieved. Once the rest of the fresh soils were dried, they were stored at 4 °C and −20 °C.

### 2.2. Experimental Design and Soil Hysicochemical Properties

A total of 200 g of soil (oven-dry basis) was placed in polycarbonate plastic containers (9 cm diameter and 12 cm height) for each experimental replicate. The initial concentrations of Fe(II) in the bottles were 0, 100, and 1000 mg kg^−1^, and the volume of deionized water was 150 mL. Sandy (S) and loamy (L) soils without the addition of FeCl_2_ were used as controls (SCK, LCK). In furtherance, sandy and loamy soils were prepared for low (L) concentrations (100 mg/kg) of FeCl_2_ as SL and LL, and for high (H) concentrations (1000 mg/kg) of FeCl_2_ as SH and LH, respectively. The laboratory simulation experiments included three replicas for each treatment, and each replicate was established in independent bottles. Next, all 18 experimental soil samples were cultivated in an incubator at 25 °C for 25 days.

The determination procedures and data of total reduced substances (TRS), Fe(II), soil organic matter (SOM), pH, and redox potential (Eh5) were described in previous publications [25,26]. To further quantify detailed soil properties, the total nitrogen, available phosphorus (TP), available potassium (AK), and total potassium (AK) were determined following standard soil testing procedures [27]. The integrated physicochemical properties of the soil are presented in Table S1.

### 2.3. Total Microbial DNA Extraction, PCR Amplification and Illumina Sequencing

Microbial DNA of eighteen soil samples was extracted from 1.0 g of each sample using an E.Z.N.A.^®^ Soil DNA Kit (Omega Biotek, Norcross, GA, USA), according to the product description. For each soil sample, modified universal bacterial primers were used to amplify the V3-V4 region: F338 (5′-ACTCCTACGGGAGGCAGCA-3′) and R806 (5′-GGACTACVSGGGTATCTAAT-3′) [28]. Each reaction contained 50 ng of DNA template, 0.2 mM dNTPs, 1.5 mM MgCl, 1.25 U Platinum Taq DNA polymerase, 2.5 mL of 10 × PCR buffer and 0.2 mM primer. The primers preceded a unique 12-base barcode (Ns) for sample recognition before mixing the amplicons. One to eight additional nucleotides were added before the barcode to offset the sequencing of primers. The thermal cycling program was as follows: 94 °C for 5 min, followed by 27 cycles of 94 °C for 50 s, 51 °C for 30 s, and 72 °C for 1 min, and a final elongation step was run at 72 °C for 10 min. Replicate PCR samples were mixed and then purified using the QIAquick PCR Purification Kit. The PCR samples were quantified and pooled with equimolar amounts before the Illumina barcodes and adaptors were ligated. The completed libraries were sequenced according to the manufacturer’s instructions on the Illumina MiSeq platform.

### 2.4. Soil Bacterial Abundance Analysis

qPCR assays were conducted to determine total bacterial biomass using the SYBR Premix Ex TaqTM Kit (Takara, Beijing, China) and iQ5 Real-Time PCR System (Bio–Rad, Hercules, CA, USA). Universal primers Eub338/Eub518 [29] were used to identify the V4 regions in the bacterial 16S rRNA genes. The PCR mix contained ten microliters each of SYBR green I and ROX dye II (Applied Biosystems, Waltham, MA, USA), 0.4 microliters of each primer, 2.0 microliters of DNA, and sterile deionized water up to a total of twenty microliters. The qPCR conditions entailed initial denaturation at 95 °C for 5 min, followed by 37 cycles of denaturation at 95 °C for 45 s, annealing at 56 °C for 45 s, and a final extension at 72 °C for 2 min. The standard samples were diluted to conduct a set of 10-fold dilutions and then used to quantify gene expression with qPCR. The standard curves were generated according to a predetermined protocol [29], and the R^2^-value exceeded 0.99. All amplifications from each individual sample were measured in triplicate.

### 2.5. Microbiome Bioinformatics and Statistical Analysis

QIIME2 (version 2020.11) was used for the 16S sequence analysis, and paired-end files were demultiplexed by the Demux plugin [30]. Then, quality control was performed on each sample by the DADA2 plugin [31]. A taxonomic arrangement of the OTUs table was classified using Greengenes-13_8 97% [32]. Furthermore, bacterial diversity and richness values were generated as alpha indices: Shannon, Simpson, ACE, and Chao. The genomic sequencing data are available in the NCBI Sequence Read Archive (SRA), and the accession number is PRJNA415121. In addition, the beta diversity of NMDS and PCoA was determined based on the OTU distance and unweighted UniFrac distance matrices. Changes in soil bacterial relative abundances were compared by ANOVA and Tukey’s HSD test at the 0.05 significance level by SPSS (version 22.0). Two-way ANOVA was used to assess Fe(II) concentrations and soil textures to determine the variables’ main effects and interactions on soil bacterial abundance and alpha diversity.

To relate the changes in bacterial community composition to environmental variables, linear regression, canonical correspondence analysis (CCA) and Mantel, Adonis and Anosim analysis were performed [33]. For the Mantel test, the soil biochemical property data were transformed into a Euclidean cumulative distance matrix, Bray–Curtis dissimilarity was used for the bacterial genera table, and 999 permutations were performed. Furthermore, to understand the effect of iron concentration on key soil biological and physicochemical characteristics, generalized linear mixed models were conducted in R using the ggplot2 package [34].

Whether microbial communities vary in function when they reside in different soils was assessed by the Phylogenetic Investigation of Communities by Reconstruction of Unobserved States (PICRUSt) [35]. This process can accurately predict bacterial community gene families with 82–95% accuracy compared to the genomic sequences of bacterial genomes [36]. The nearest sequenced taxon index (NSTI) was also used as an evaluation measure to describe organisms’ novelty in an OTUs table compared with previously sequenced genomes [35]. At level 3 of KEGG (Kyoto Encyclopedia of Genes and Genomes) Orthology, the predicted bacterial gene abundances were analyzed. We analyzed the top 19 KEGG pathways and determined the changes in carbon, nitrogen and sulfur pathways that occurred in soil communities.

### 2.6. Co-Occurrence Network Analysis

Utilizing network analysis, the co-occurrence patterns of bacterial taxa were illustrated with the R package igraph [37,38]. We adopted Spearman’s correlation test to obtain a strong correlation (*r* > |0.9|, *p* < 0.01) between the 30 most abundant genera of bacterial communities. Afterward, we used Cytoscape version 3.8.0 [39,40] to visualize the network structure. The size of each node represents the concentration of bacterial genera in the habitat. Node color coded by the taxonomy category and the shape of the node is divided into chemoautotrophic and other taxa. The width of the links represents how closely the microbes are related to each other. At the same time, a network analyzer was utilized to calculate the obtained network topology parameters to represent the co-occurrence relationship between genera [41].

## 3. Results

### 3.1. Soil Bacterial Abundance and Community Diversity

**Figure 1:**
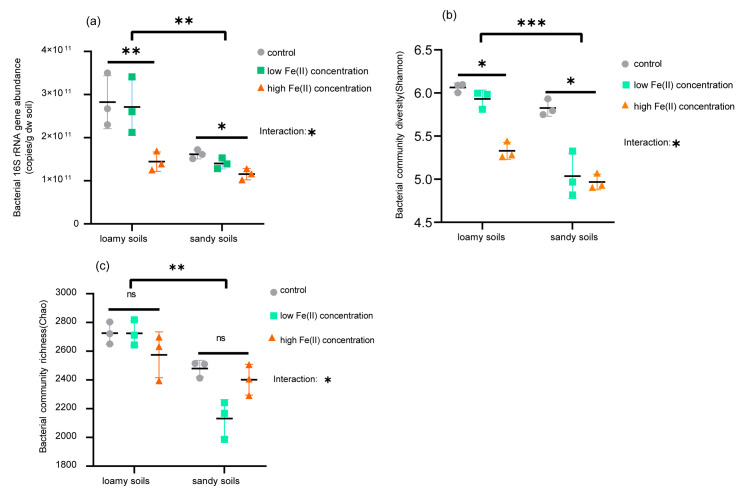
The bacterial abundance and diversity in different soil treatments; 16S rRNA gene copy numbers derived from qPCR (**a**) and indices of alpha diversity shown as Shannon (**b**) and Chao (**c**). Significant differences between Fe(II) concentrations and soil textures were determined by two-way ANOVA marked by different capital letters. The symbols *, **, and *** are used to show statistical significance at the 0.05, 0.01, and 0.001 level, respectively. ns represents no statistical significance at the *p* = 0.05 level.

High-quality 16S rRNA gene sequences of 760 and 672 V3-V4 were obtained from 18 soil samples, with 3200 OTUs in total. The rarefaction curves based on OTUs were smooth, indicating that the sequence data were reliable for analysis (Figure S1). The total sequence reads, soil bacterial abundance and alpha diversity in the different treated soils are summarized in Table S2. As shown in Table S2, the interaction of Fe(II) concentrations and soil textures significantly affected the soil abundance, diversity and richness of bacterial communities. The copy numbers of the bacterial 16S rRNA gene varied from 1.16 × 10^11^ (SH) to 2.67 × 10^11^ copies g^−1^ (LH) across all soil samples. The soil bacterial abundance of LCK and LL soils was significantly higher than that of LH and all sandy soils (Figure 1a). In addition, high Fe(II) concentrations (SH) significantly reduced the bacterial abundance across sandy soils. The t-test ANOVA suggested remarkable differences in 16S rRNA gene abundance between the two textured soils, with higher values observed in loamy soils than sandy soils. In loamy soils, the diversity index (Shannon) of LCK and LL (Figure 1b) was significantly higher than that of LH (*p* < 0.05). For sandy soils, the Shannon index of SCK (Figure 1c) was significantly greater than that of SL and SH. In contrast, Fe(II) concentrations had no clear effects on bacterial richness (Chao) between treatments (*p* > 0.05). Additionally, the t-test ANOVA showed that the Shannon and Chao indices of loamy soils were notably higher than those of sandy soils.

**Figure 2:**
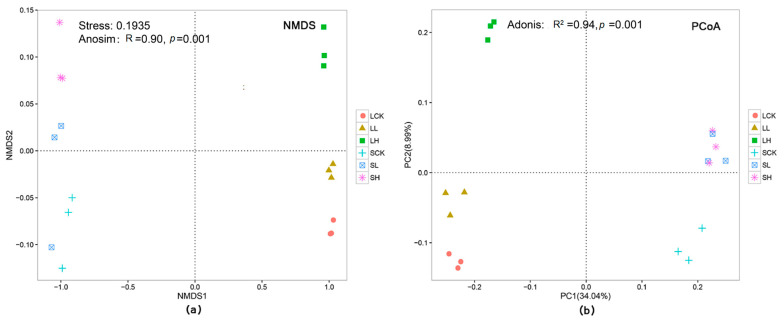
β-diversity of microbial community structure across the different soil treatments. (**a**) Nonmetric multidimensional scaling (NMDS) plot of bacterial Bray–Curtis dissimilarity. (**b**) Principal Coordinate Analysis (PCoA) of 16S rRNA diversity in the paddy soils based on Unweighted UniFrac distances. Each point represents one metagenomic sample (*n* = 18). Data representing relative variable importance (*R*^2^) and significance (*p*) calculated by PERMANOVA (Adonis) are displayed.

The NMDS analysis at the OTU level illustrated the differences in bacterial β-diversity based on the Euclidean distance (Figure 2a). The NMDS plot demonstrated that the same treatment triplicates were not close, showing good experimental repeatability. Of all treated characteristics considered, iron concentrations and soil textures had distinct effects on the bacterial community composition. Additionally, differences in the composition among treatments were significant (*R* = 0.90, *p* = 0.001, Figure 2a), according to ANOSIM. As shown in Figure 2b, the unweighted UniFrac-based PCoA showed that the compositions of different soil sample sequences were separated. The distance between loamy and sandy soil samples increased along with the Fe(II) concentration. In the loamy soil treatments, LH was far from LCK and LL, while in the sandy soil treatments, SL and SH were relatively close, indicating that SL and SH could not be clearly separated. According to the Adonis, the bacterial β-diversity among treatments was also significant (*R*^2^ = 0.94, *p* = 0.001, Figure 2b). Two models, namely, NMDS and PCoA, yielded a similar trend, and both of them could clearly reveal the difference and similarity in bacterial β-diversity among the groups.

### 3.2. Analysis of Soil Bacterial Community Structure under Different Fe(II) Concentrations

**Figure 3:**
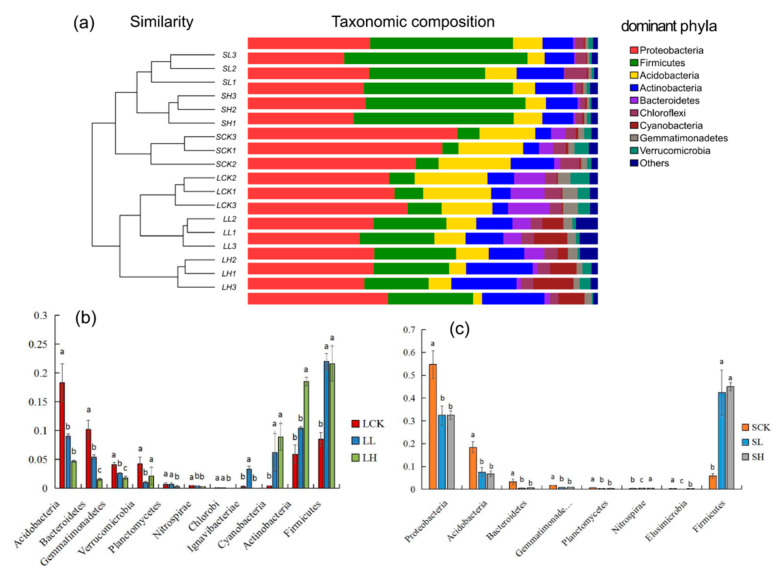
(**a**) Changes in the relative abundances of bacterial dominant phyla. (**a**) Clustering tree of bacterial dominant phyla, and others includes phyla below 0.1% of relative abundance and the unclassified phyla. It is significantly different in loamy soil (**b**), sandy soil (**c**). Different letters to show statistical significant differences by an ANOVA analysis (*p* < 0.05, *n* = 3).

The UniFrac clustering tree (Figure 3a) showed that there were obvious differences in the relative abundance of bacteria among the six treatments, in which LH and SCK had the farthest sequence similarity. Further, the bacteria in the same treatment samples could be closely clustered. Nine predominant phyla were mainly populated by Proteobacteria, Firmicutes, and Acidobacteria, and they occupied more than 70% of the bacterial community. Interestingly, the relative abundance of seven phyla decreased significantly with the addition of Fe(II). However, three increased significantly (Figure 3b). The relative abundance of five phyla in the sandy soils decreased significantly with the addition of Fe(II), and only one increased considerably (Figure 3c).

**Figure 4:**
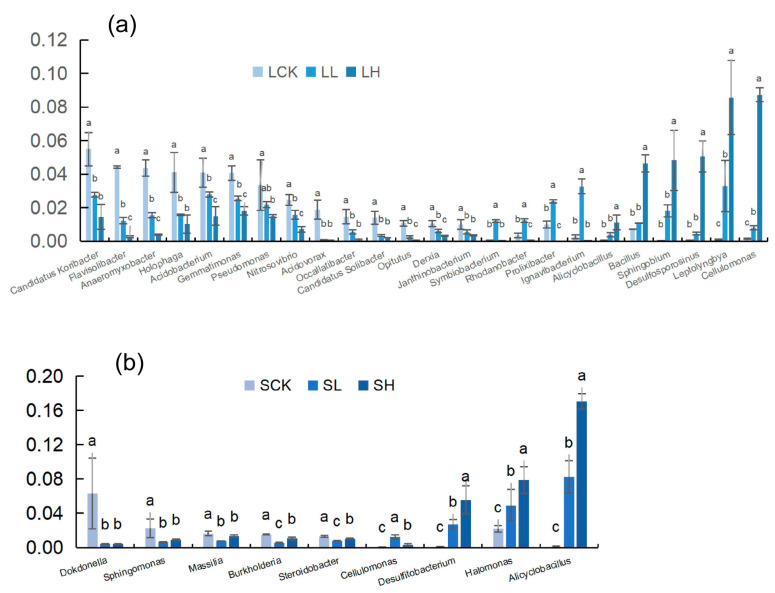
Changes in the relative abundances of bacterial dominant genera (relative abundance ≥ 1%) under different treatments in loamy (**a**) and sandy (**b**) soils. Different letters to show statistical significant differences by an ANOVA analysis (*p* < 0.05, *n* = 3).

The dominant bacterial genera in the different samples are shown in Figure 4. The relative abundance of thirteen genera in loamy soils decreased significantly with increasing Fe(II) concentrations, and these taxa were the most abundant in the control. Nevertheless, six genera showed the opposite trend. Surprisingly, we found that four genera were most predominant at low Fe(II) concentrations (Figure 4a). For sandy soils, five genera were richer in the SCK. Furthermore, three genera were significantly more prevalent in the SH treatment than in the other treatments (Figure 4b).

In our results, the dominant genera in the submerged environment of the two textured control soils were mainly aerobic bacteria. With the addition of Fe(II), anaerobic bacteria increased from 9.23% (LCK) to 17.22% (LH), but LL only accounted for 5.51%. In sandy soils, anaerobic bacteria increased in succession along with the concentration of Fe(II), from 5.93% (SCK) to 20.33% (SL) and 32.47% (SH). These anaerobic or facultative anaerobes include *Alicyclobacillus*, *Halomonas*, *Holophaga*, *Cellulomonas*, *Desulfitobacterium*, *Anaeromyxobacter*, and *Desulfosporosinus* (Table S3). According to their mode of nutrition (Table S4), we found that there were four typical chemoautotrophic bacteria (*Alicyclobacillus*, *Leptolyngbya*, *Nitrosovibrio*, and *Desulfosporosinus*). In loamy soils, chemoautotrophic bacteria increased from 2.60% (LCK) to 5.55% (LL) and 15.39% (LH), and in sandy soils, chemoautotrophic bacteria increased from 3.28% (SCK) to 11.29% (SL) and 18.95% (SH).

### 3.3. Relationships between Bacterial Communities and Soil Physicochemical Properties

**Figure 5:**
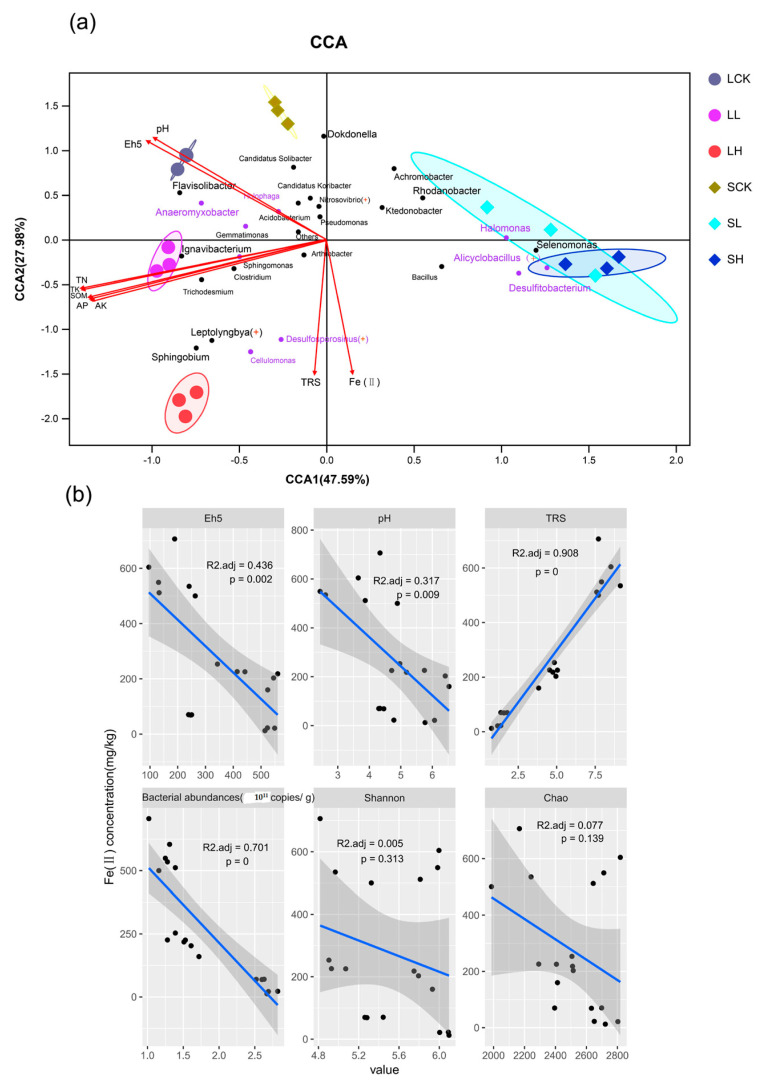
Relationships between bacterial communities and soil physicochemical properties. (**a**) Canonical correspondence analysis (CCA) of taxa (relative abundance of the dominant genera represented by more than 1% in at least one sample) and eight environmental parameters (pH, Eh5, TRS, AP, AK, TK, TN and SOM). TRS, total reduced substances; AP, available phosphorus; AK, available potassium; TN, total nitrogen; SOM, soil organic matter (**a**). Node colored by the category of anaerobic or other taxa, and the shape of the node is divided into chemoautotrophic and other taxa. The dot and genus name colored by purple represent anaerobic bacteria, while the genus name marked “+” represents chemoautotrophic bacteria. (**b**) General linear mixed models: correlations between Eh5, pH, TRS, total bacterial abundances (16S rRNA gene abundances), bacterial community diversity (Shannon index), bacterial community richness (Chao index) with the Fe(II) concentration. Lines in all panels show linear regression fitting.

We performed canonical correspondence analysis (CCA) to illustrate the influence of environmental factors (pH, Eh5, TRS, AP, AK, TK, TN, and SOM) (Figure 5a and Table S5) on the soil microbial community structure and compositions. Moreover, the Mantel test revealed significant (*p* < 0.05, 999 permutations) relationships between bacterial community compositions and most environmental factors, except TK (Table S5). As noted above, the soil bacterial community structure was significantly (*p* < 0.05) affected by the soil texture type and the Fe(II) concentration, based on Adonis analysis. CCA indicated that 75.57% of the total variance within seven environmental variables was explained by the first (47.59%) and second (27.98%) ordination axes (Figure 5a). The first axis separated the sandy soils from the loamy soils.

Furthermore, all the samples were clearly distinguished by different treatments on the second axis due to the Fe(II) concentration, especially in loamy soil samples that occupied the most bacterial community. Soil organic matter and nutrients (including AP, AK, TK, TN and SOM) showed significant positive correlations, and they had a notably positive correlation with the bacterial community (especially LL) in loamy soils. The SL and SH treatments grouped together and correlated with the dominant bacterial genera of *Alicyclobacillus*, *Selenomonas*, *Desulfitobacterium*, and *Halomonas*. In addition, soil pH and Eh5 were strongly correlated with the SCK treatment.

To explore the effect of iron concentration on key biological and physicochemical indices of soil, general linear mixed models were used to build the relationship (Figure 5b). We found that the Fe(II) concentration had a strong positive correlation with TRS (*R*^2^ = 0.908, *p* = 0). On the other hand, the concentration of Fe(II) negatively correlated with Eh5 (*R*^2^ = 0.436, *p* = 0.002) and pH (*R*^2^ = 0.701, *p* = 0.009). Notably, the concentration of Fe(II) had a strong negative correlation with total bacterial abundance (*R*^2^ = 0.317, *p* = 0). However, there was a nonsignificant relationship between the concentration of Fe(II) and the diversity of the bacterial communities (Shannon index), as was the richness of the bacterial communities (Chao index).

### 3.4. Potential Bacterial Co-Occurrence Networks of Fe(II)-Mediated Interactions

**Figure 6:**
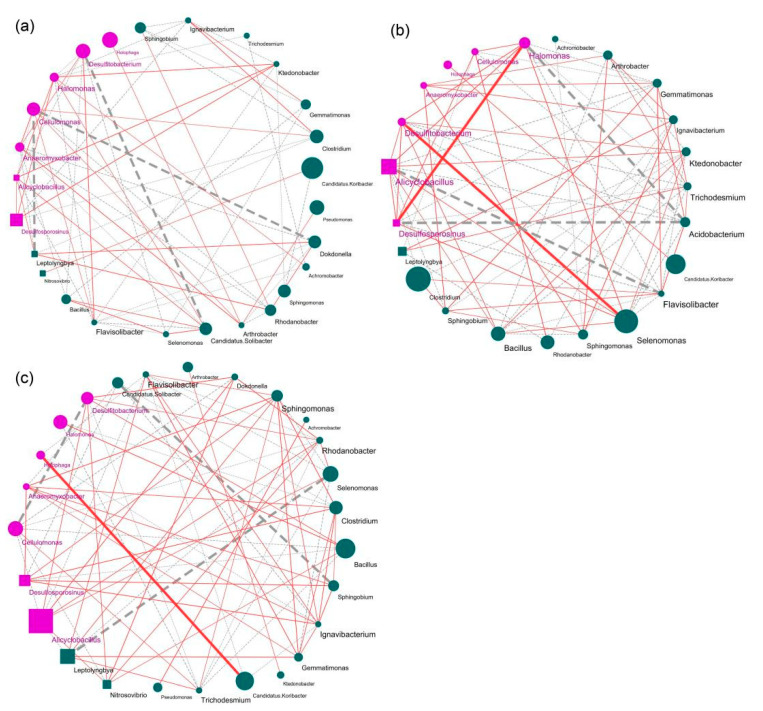
Co-occurrence network diagram of soil top 30 bacterial genera between different Fe(II) concentration systems, and we classify microorganisms according to their type of oxygen demand and mode of nutrition. We define the treatment of LCK and SCK as an integrated system (LSCK) (**a**). Similarly, Fe (II) low additive treatment is LSL (**b**); Fe (II) high additive is an integrated system of LSH (**c**). We construct separate network diagrams based on an integrated system. Based on Spearman correlation, Cytoscape was used to construct bacterial co-occurrence network. Correlation is shown as edge (positive correlation = red; Negative correlation = gray), correlation coefficient *r* > |0.9|, and *p* < 0.01. Node colored by the category of anaerobic or other taxa, and the shape of the node is divided into chemoautotrophic and other taxa. The size of nodes is positively correlated with relative abundance of genus. The width of the link represents the correlation strength between microbes at genus level, to find the keystone genera within networks.

We defined LCK and SCK as an integrated system (LSCK), and we considered LSL and LSH to be similarly integrated. The co-occurrence networks of the three systems were constructed by using Cytoscape software to clarify the responses of different Fe(II) concentrations to soil microbial interactions. Network analysis was constructed using the 30 most abundant genera of soil bacteria (Figure 6).

Eleven parameters representing the network topology parameters were estimated (Table S6). The results showed that the total links, positive links, network density, network diameter, and average neighbors all increased along with the Fe(II) concentration. In particular, the bacterial network of LSL is the most complex and balanced, and it holds the highest topological properties of total links (78) and network density (0.308); however, the simplest network (LSCK) has links and network density of only 66 and 0.203, respectively. Although the addition of Fe(II) greatly changed the co-occurrence patterns of soil bacteria, there was no apparent difference between high and low Fe(II) concentrations.

We further focused on the network of anaerobic and autotrophic bacteria and found that the total links and positive links of anaerobic bacteria in LSL (purple nodes) were the highest. Surprisingly, these characteristics of autotrophic bacteria (square nodes) increased sharply with the concentration of Fe(II). Based on the greatest correlations between microbes, we sought to find the keystone genera within each network. The higher the correlation between the genus and other taxa was, the thicker the links in the network were. In the LSCK system, *Desulfitobacterium* was negatively correlated with *Candidatus*, *Cellulomonas* and *Dokdonella*. In the LSL system, there were four bacterial genera with the most negative links: *Desulfitobacterium*, *Acidobacterium*, *Halomons*, and *Alicyclobacillus*. In addition, *Desulfitobacterium* was positively correlated with *Halomonas*, *Desulfosporosinus* and *Selenomonas*. In the LSH system, there were multiple bacterial genera with the most negative links, including *Cellulomonas*, *Leptolyngbya*, *Desulfitobact*, *Sphingobrium*, *Selenomonas*, and *Candidatus Solibacter*. In addition, *Halomonas* was positively correlated with *Candidatus Koribacter*. These keystone genera are mostly anaerobic and facultative anaerobic bacteria, which have significant interactions with the addition of Fe(II).

### 3.5. Prediction of Bacterial Metabolic Functions Based on PICRUSt

**Figure 7:**
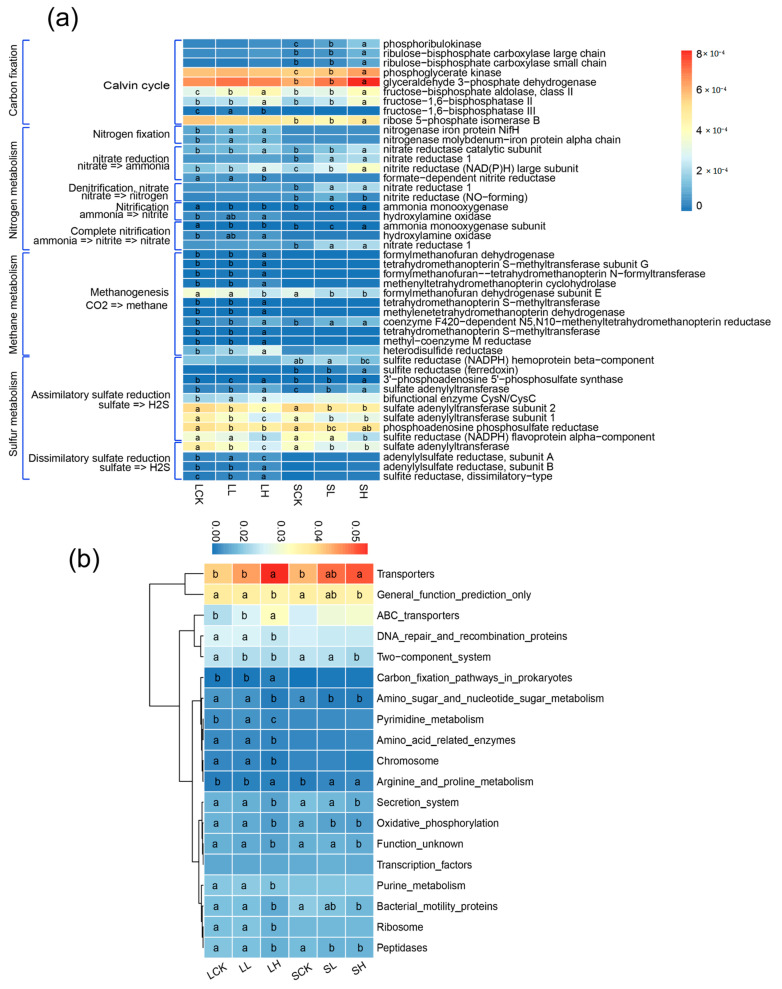
Predicted functions of top 19 of KEGG pathway in different soil treatments. (**a**) Energy metabolic pathways at second level, (**b**) variety of energy metabolic pathways at third level. Different letters to show statistical significant differences by an ANOVA analysis (*p* < 0.05, *n* = 3).

We attempted to predict the potential functional genes associated with different Fe(II)-treated soils based on bacterial OTUs. All of the treatments showed strong NSTI values of 0.114 ± 0.006–0.159 ± 0.002, which means that these values provide a dataset suitable for PICRUSt prediction. In the predicted metagenomes, 19 categories at level 3 of the KEGG Orthology groups (KOs) are shown in Figure 7. Along with different Fe(II) concentrations, the transporter function and arginine and proline metabolism function were promoted to some extent in both textured soils. In contrast, some functions, such as amino sugar and nucleotide sugar metabolism and bacterial motility proteins, were inhibited (Figure 7a). We investigated some energy metabolic pathways and showed differences in the two textured soils (Figure 7b). In loamy soils, some Calvin cycle, nitrogen fixation and methane generation metabolism pathways were promoted to some extent, but some sulfate reduction pathways were partially inhibited. In sandy soils, the Calvin cycle and nitrate reduction pathways were partially promoted, while part of the assimilatory sulfate reduction pathway was inhibited.

## 4. Discussion

Cultivated rice is an annual herb that evolves from a semiaquatic ancestor and mostly grows in flooded wetlands. Due to farmland management practices, including flooding and drainage regimes, redox potential oscillations are associated with the balance of Fe(III)/Fe(II) coupled reactions [42]. Iron is a vital limited resource in soil, and its effect on the microbial community structure and ecological function remains unknown [43]. Therefore, the roles of microorganisms in energy and material conversion have become an important issue [44,45]. In this study, we explored the effects of Fe(II) concentration on the bacterial community, co-occurrence network and function of two textured paddy soils.

Judging from qPCR results, the abundance of soil bacteria in loamy soils was remarkably higher than that in sandy soils, which was consistent with our previous result on the *cbbM* gene [25]. This indicated that soil with more available nutrients is conducive to carbon sequestration functional microorganism and total bacterial growth. In addition, Fe(II) at high concentrations significantly reduced the absolute abundance of bacteria in the two paddy textured soils. Recent research found that copper at high concentrations has a more lasting effect on the bacterial community in swine manure compost, and the decrease in bacterial abundance and ARGs was positively correlated with the exchangeable Cu concentration [24]. These consistent trends suggest that the metal ion concentrations play a crucial role in regulating microbial populations.

Based on the alpha diversity, we found that Fe(II) at high concentrations in loamy soils significantly decreased the bacterial community diversity, which was consistent with the total bacterial abundance. By comparison, for sandy soils, the addition of Fe(II) significantly reduced soil bacterial diversity. The possible reason for this phenomenon may be different buffering capabilities in the two textured soils. To date, several studies have confirmed that loamy soils have a more robust buffering capability than sandy soils when exogenous substances enters or the external environment changes [17,19]. Our experiment found that when the same concentration of Fe(II) was added, the changes in TRS, Eh5 and pH in loamy soils were milder than those in sandy soils. Moreover, bacterial diversity in sandy soils was more sensitive than that in loamy soils after Fe(II) addition. Thus, soil textures and Fe(II) concentrations may be the main factors shaping bacterial community structures [46,47].

The analysis of the soil bacterial community at the genus level revealed that Fe(II) directly shaped the abundance of anaerobic and chemoautotrophic bacteria. In LCK and SCK, aerobic genera (*Pseudomonas* [48], *Sphingomonas* [49], *Nitrobacter* [50] and *Acidovorax* [51,52] were dominant, and they were significantly inhibited with the Fe(II) addition. Therefore, we assume that oxygen depletion caused by Fe(II) oxidation further inhibits the abundance of these genera. According to the statistics of the total relative abundance of anaerobic bacteria in separate treatments, we found that they all increased significantly with the Fe(II) addition, and the most significant increase was from 6.67% (SCK) to 32.77% (SH) in sandy soils. This can reasonably explain the conjecture previously mentioned. At the same time, we found that the total relative abundance of four typical chemoautotrophic bacteria (*Alicyclobacillus* [53], *Leptolyngbya* [54], *Nitrosovibrio* [50], and *Desulfosporosinus* [55]) in the two textured soils increased significantly with the Fe(II) concentration. For these chemoautotrophic bacteria, Fe(II) can be used as an alternative electron donor. In our experiment, the addition of a high Fe(II) concentration significantly increased TRS and decreased the soil oxygen content, providing a suitable habitat for chemoautotrophic and anaerobic bacteria.

Canonical correspondence analysis also supports the conclusion that the Fe(II) addition has a greater influence on sandy soil properties than loamy soils (Figure 5). Thus far, it has been demonstrated that soil physicochemical and biological characteristics play essential roles in the microbial community structure, such as organic matter, Eh5, and pH [56] In this study, Eh5, TRS, AP, AK, TN and SOM were also the key factors affecting the soil bacterial community after the Fe(II) addition. Specifically, it suggests that these bacterial taxa are more likely to inhabit loamy soils and that their growth is further promoted under appropriate iron concentrations. As a reducing substance, the introduction of Fe(II) will increase the total reducing substances in soil. Second, the addition of Fe(II) was negatively correlated with Eh5 and pH but had no significant relationship with total bacterial abundance, bacterial diversity or richness. Individual studies have shown that reactive oxygen produced by chemical oxidation of Fe(II) is biotoxic [57] and can kill microorganisms such as *Escherichia coli* [58,59]. Notably, it has been suggested that cells of different functional microorganisms exhibit different adsorption properties for Fe(II), and resistance to reactive oxygen species [60].

Previous studies have found that resource competition is the primary factor affecting microbial interactions [61]. We further analyzed the ecological networks of specific bacteria in soil systems with different Fe(II) concentrations. The results showed that the addition of Fe(II) greatly promoted the network structure of soil bacteria, and the network of total bacteria and anaerobic bacteria in the low- and high-concentration Fe(II) systems was more complex; in particular, the number of positive links increased gradually with the increasing concentration of Fe(II). In our experiment, the keystone genera of the ecological network keystone genera were mainly dominated by some anaerobic and autotrophic bacteria. Among them, *Desulfitobacterium* [62] is an excellent candidate for developing anaerobic bioremediation processes, while *Cellulomonas* can scavenge a variety of heavy metal ions, such as Cr(VI) and Fe(III). *Alicyclobacillus* [53] exists widely in acidic and thermal environments such as mines and can oxidize ferrous ions and sulfide ores, promoting autotrophic growth. *Desulfosporosinus* [55] can generate energy by reducing sulfate to sulfide and has a critical control function in methane production. We found that *Desulfosporosinus* plays a critical role in the environment of Fe(II) addition.

More surprisingly, the association of autotrophic bacteria in the symbiotic network surged with the Fe(II) concentration. According to these results, we confirmed that the addition of Fe(II) would make the interaction between soil anaerobic and autotrophic bacteria more complex and modular, thus driving the construction of the whole microbial network. Previous research has shown that the total metabolites secreted by some beneficial bacteria can mediate microbial interactions, serving as promoters under iron-rich conditions [63]. Moreover, a recent study explored the effect of dissolved oxygen on the denitrification characteristics of mixed bacteria and found that keystone taxa (such as *stappia* and *Pseudomonas*) contributed to the high TN removal efficiency through the co-occurrence network [64]. Our results also suggest that anaerobic bacteria can play a decisive role in the microecological network.

The microbial-mediated iron cycle strongly affects the carbon and nitrogen cycles in soil and the degradation of organic/inorganic pollutants [65]. According to PICRUSt analysis, transport increased with Fe(II) concentrations in two textured soils. We hypothesized that after Fe(II) addition, the environment and competition pattern changed, eliminating the genera that could not adapt to the changes. The remaining genera increased some metabolic pathways that are beneficial to their survival. In our study, ABC transport in LH was significantly increased, which may be because ABC transporters constitute large amounts of membrane proteins and can transport diverse substrates, such as metal(loid)s and secondary metabolites [66]. Fe(II) addition significantly reduced the assimilatory sulfate reduction metabolism pathway in two textured soils, which is quite the opposite of previous research [67]. This result suggests that Fe(II) addition inhibits the synthesis of organic sulfur compounds from sulfates. As shown in Figure 6b, part of the dissimilatory sulfate reduction process increased along with the Fe(II) concentration in loamy soils. This result requires further investigation since the increase is usually associated with toxic hydrogen sulfide gas in anoxic environments [68]. High Fe(II) concentrations significantly enhanced the methane metabolic pathway. There are relatively few findings [69,70] that have revealed that the addition of ferric iron reduces methane production; ferrous iron will be converted into ferric iron under drainage conditions, promoting the regeneration of ferric iron. Therefore, periodic drainage in paddy soil helps to reduce methane emissions. Our study found that Fe(II) addition inhibited the relative abundance of the genus *Anaeromyxobacter* competing with the intermediates of methanogenic pathways. Additionally, it promoted the growth of *Desulfosporosinus* and *Desulfitobacterium*, which produce methane through intermediate products. In sandy soils, a high Fe(II) concentration was conducive to the Calvin cycle. Using the KEGG pathway query, we found that the partial pathways were associated with the reductive pentose phosphate cycle model, reducing CO_2_ emissions through carbon dioxide fixation. Moreover, the nitrate reduction pathway was promoted after Fe(II) addition. Therefore, increased nitrite is beneficial to the growth of the genera *Cellulomonas* and *Halomonas* under anaerobic conditions. According to researchers [25,71], irrigated paddy ecosystems could store substantial carbon in the soil. From the perspective of microbial metabolic characteristics, Fe(II) promotes methanogenesis and the Calvin cycle, and the microbial community could adapt to the dynamic environment by forming a functional maintenance mechanism.

To conclude, our research provides novel ecological insight into the soil co-occurrence patterns and function in Fe(II)-added paddy soils. Our findings help in the understanding of bacterial communities and their functions in the redox change region of the Earth environment, and provide the basis for agriculture and soil sustainability. However, the effects of Fe(II) on the ecological functions of paddy soil, both the rice rhizosphere and the endophyte, need to be evaluated. In addition, with the complete application of high-throughput sequencing technology, it is necessary to carry out iron carrier and multiomics research in the future to explain the molecular mechanism of Fe(II) oxidation and Fe(III) reduction in these contexts.

## 5. Conclusions

The impacts of Fe(II) concentrations on the bacterial communities and functions of two textured paddy soils were explored by laboratory experiments. The results showed that Fe(II) addition changed the original environmental conditions, resulting in changes in soil physio-chemical properties. Although a high Fe(II) concentration could significantly reduce the total bacterial abundance and community diversity, the bacterial abundance in loamy soils was generally higher than that in sandy soils. In addition, the total relative abundance of chemoautotrophic and anaerobic bacteria increased significantly with the concentration of Fe(II), and the network among them and the dominant bacteria was closer after Fe(II) addition. Simultaneously, the metabolic pathway showed that the addition of Fe(II) promoted the activity of the methane pathway, Calvin cycle, and nitrate reduction pathway, which were mediated by the enrichment and interrelation of autotrophic and anaerobic bacteria.

## Data Availability

The data and results of this study are available upon reasonable request. Please contact the main author of this publication.

## Supplementary Materials

The following are available online at https://www.mdpi.com/article/10.3390/microorganisms10030547/s1, Figure S1: Rarefaction curves for 16S rRNA gene diversity. Curves are colored according to soil treatments; Table S1: Soil physicochemical properties in different Fe(II) treatments, Table S2:Soil Bacterial Abundance and α-diversity in soil samples, Table S3: The members of Anaerobic bacteria genera observed in in paddy soils, Table S4: The members of Chemoautotrophic bacteria genera observed in in paddy soils, Table S5: Mantel test showing the correlations between soil properties and the community com-position of bacteria in sandy soils, Table S6: Topological properties of Correlation network diagram of soil bacterial communities at genus level in soil systems with different Fe (II) concentrations
