# Supplementary material for: Fe(II) Addition Drives Soil Bacterial Co-Ocurrence Patterns and Functions Mediated by Anaerobic and Chemoautotrophic Taxa
Source: Microorganisms. 2022 Mar 2;10(3):547. doi: 10.3390/microorganisms10030547 (PMC8950066; doi:10.3390/microorganisms10030547)
Supplement: Supplementary file 1 [file microorganisms-10-00547-s001.zip › microorganisms-1583761-supplementary.pdf]

SUPPLEMENT

Table S1

Soil physicochemical properties in different Fe(Ⅱ) treatments. Total reduced substances (TRS), Fe(Ⅱ), Soil organic matter (SOM), pH and Eh5 were determined and cited from our previous studies[26]. The total nitrogen (TN), Available phosphorus (TP), Available potassium(AK), and total potassium(AK) were further determined with the same soil treatments.

Treatments	TN (g/kg)	AP (g/kg)	TK (g/kg)	AK (mg/kg)	SOM (g/kg)	TRS (cmol/kg)	Fe (Ⅱ) (g/kg)	Eh5	pH
LCK	1. 68±0. 02	0. 65±0. 05	12. 49±0. 52	222.69±30b	13.29±0. 43	4. 51±0. 60b	194±30b	543±18a	6. 04±0. 75a
LL	1. 69±0. 03	0. 71±0. 03	12. 51±0. 24	226.49 ±30b	14. 12±0. 31	4. 81±0. 26b	235±16b	400±51b	5. 14±0. 54b
LH	1. 60±0. 11	0. 67±0. 03	11. 88±0. 24	239.20 ±30b	13. 28±0. 50	8. 81±0. 82a	580±110a	231±38c	3. 95±1. 19b
SCK	1. 00±0. 03	0. 49±0. 02	6. 50±0. 36	55.51±30b	7. 87±0. 20	1. 11±0. 31b	18±6b	529±18a	5. 54±0. 68a
SL	0. 99±0. 05	0. 50±0. 02	6. 57±0. 31	53.16±30b	8. 49±0. 33	1. 58±0. 21b	70±1b	245±6b	4.37±0. 08ab
SH	0. 98±0. 04	0. 48±0. 02	6. 47±0. 46	52.16 ±30b	7. 85±0. 33	8. 01±0. 47a	555±46a	120±21c	3. 23±0. 77b

Note: Values are means ± standard deviation, different soil treatments (n=3). Means followed by a different letter for a given factor are significantly different in same soi texture ($P < 0.05$; Analysis of ANOVA).

Table S2.

Soil Bacterial Abundance and α-diversity in soil samples.

Treatments	Reads	OTUs	Coverage	Richness indices		Diversity indices		Soil Bacterial Abundance
				Chao	ACE	Shannon	Simpson	16S rRNA genes(copies/g)
LCK	31536	2751	0. 9792	2725±62^{a}	2728±47^{a}	6. 10±0. 04^{a}	0. 0066±0. 0006^{b}	2.82E+11^{a}
LL	36812	2790	0. 9827	2725±72^{a}	2734±54^{a}	5. 93±0. 09^{a}	0. 0090±0. 0013^{b}	2.71E+11^{ab}
LH	38430	2625	0. 9839	2575±130^{a}	2576±94^{a}	5. 33±0. 08^{b}	0. 0226±0. 0032^{a}	1.44E+11^{b}
SCK	34144	2609	0. 9833	2479±47^{A}	2454±105^{A}	5. 82±0. 08^{A}	0. 0113±0. 0024^{B}	1.61E+11^{A}
SL	32101	2299	0. 9825	2132±107^{B}	2174±132^{A}	5. 04±0. 21^{B}	0. 0289±0. 0071^{A}	1.40E+11^{AB}
SH	39909	2533	0. 9851	2401±87^{AB}	2442±99^{A}	4. 97±0. 07^{B}	0. 0368±0. 0030^{A}	1.15E+11^{B}

Note: All values are mean ± standard deviation in different soil treatments. Columns in the same soil type with different letters were significantly different according to an ANOVA analysis ($P<0.05$, n=3).

Table S3.

The members of Anaerobic bacteria genera observed in in paddy soils.

Genus	LCK	LL	LH	SCK	SL	SH
Alicyclobacillus	0.0002	0.0040	0.0112	0.0012	0.0820	0.1693
Halomonas	0.0041	0.0050	0.0013	0.0219	0.0490	0.0783
Holophaga	0.0410	0.0158	0.0102	0.0350	0.0163	0.0174
Cellulomonas	0.0016	0.0080	0.0869	0.0000	0.0126	0.0035
Desulfitobacterium	0.0016	0.0021	0.0084	0.0005	0.0271	0.0550
Desulfosporosinus	0.0002	0.0046	0.0503	0.0007	0.0162	0.0012
Anaeromyxobacter	0.0436	0.0155	0.0040	0.0074	0.0020	0.0030
total	0.0923	0.0551	0.1722	0.0667	0.2053	0.3277

Table S4.

The members of Chemoautotrophic bacteria genera observed in in paddy soils.

Genus	LCK	LL	LH	SCK	SL	SH
Alicyclobacillus	0.0016	0.0075	0.0869	0.0012	0.0820	0.1693
Leptolyngbya	0.0016	0.0022	0.0084	0.0025	0.0006	0.0016
Nitrosovibrio	0.0311	0.0363	0.0452	0.0285	0.0140	0.0174
Desulfosporosinus	0.0034	0.0118	0.0006	0.0007	0.0162	0.0012
total	0.0377	0.0578	0.1410	0.0328	0.1129	0.1895

Table S5.

Mantel test showing the correlations between soil properties and the community com-position of bacteria in sandy soils.

Environmental Factor	r^2	Pr(>r)
pH	0.1814	0.019
Eh5	0.4313	0.001
TRS	0.2406	0.015
AP	0.3573	0.003
AK	0.541	0.008
TK	0.203	0.253
TN	0.43	0.002
SOM	0.4118	0.001
Fe	0.1535	0.028

Table S6.

Topological properties of of Correlation network diagram of soil bacterial communities at genus level in soil systems with different Fe (II) concentrations.

Parameters	LSCK	LSL	LSH
nodes	26	23	26
Total links	66	78	77
Positive links	29	42	47
Negative links	37	36	30
Clustering coefficient	0.412	0.563	0.569
Network density	0.203	0.308	0.298
Shortest paths	650(100%)	422(83%)	466(71%)
Network diameter	7	4	4
Average neighbors	5.077	6.783	6.578
Links of Anaerobic bacteria	46(Positive:23)	50(Positive:30)	42(Positive:24)
Links of Chemoautotrophic bacteria	20(Positive:9)	24(Positive:13)	28(Positive:20)

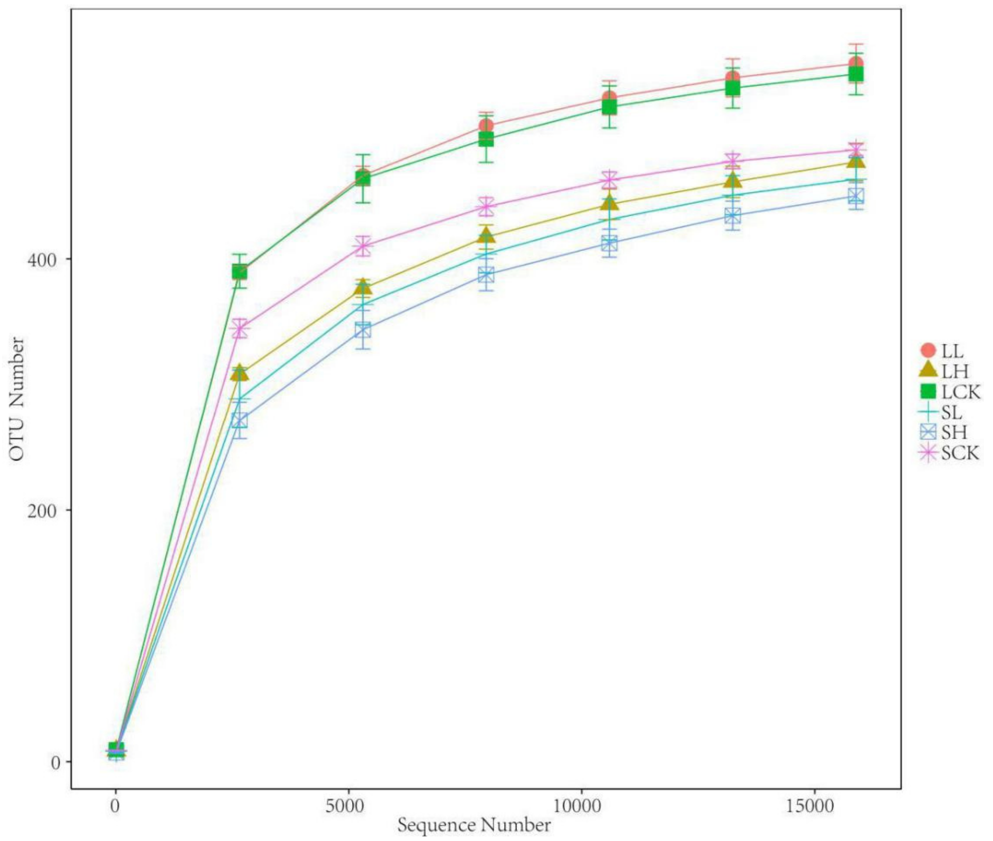

Fig S1. Rarefaction curves for 16S rRNA gene diversity. Curves are coloured according to soil treatments.
